# Poincaré and SimBio: a versatile and extensible Python ecosystem for modeling systems

**DOI:** 10.1093/bioinformatics/btae465

**Published:** 2024-07-30

**Authors:** Mauro Silberberg, Henning Hermjakob, Rahuman S Malik-Sheriff, Hernán E Grecco

**Affiliations:** Universidad de Buenos Aires, Facultad de Ciencias Exactas y Naturales, Departamento de Física, Buenos Aires 1426, Argentina; CONICET – Universidad de Buenos Aires, Instituto de Física de Buenos Aires (IFIBA), Buenos Aires 1426, Argentina; European Bioinformatics Institute, European Molecular Biology Laboratory (EMBL-EBI), Wellcome Genome Campus, Cambridge, CB10 1SD, United Kingdom; European Bioinformatics Institute, European Molecular Biology Laboratory (EMBL-EBI), Wellcome Genome Campus, Cambridge, CB10 1SD, United Kingdom; European Bioinformatics Institute, European Molecular Biology Laboratory (EMBL-EBI), Wellcome Genome Campus, Cambridge, CB10 1SD, United Kingdom; Department of Surgery and Cancer, Faculty of Medicine, Imperial College London, London, SW7 2AZ, United Kingdom; Universidad de Buenos Aires, Facultad de Ciencias Exactas y Naturales, Departamento de Física, Buenos Aires 1426, Argentina; CONICET – Universidad de Buenos Aires, Instituto de Física de Buenos Aires (IFIBA), Buenos Aires 1426, Argentina

## Abstract

**Motivation:**

Chemical reaction networks (CRNs) play a pivotal role in diverse fields such as systems biology, biochemistry, chemical engineering, and epidemiology. High-level definitions of CRNs enables to use various simulation approaches, including deterministic and stochastic methods, from the same model. However, existing Python tools for simulation of CRN typically wrap external C/C++ libraries for model definition, translation into equations and/or numerically solving them, limiting their extensibility and integration with the broader Python ecosystem.

**Results:**

In response, we developed Poincaré and SimBio, two novel Python packages for simulation of dynamical systems and CRNs. Poincaré serves as a foundation for dynamical systems modeling, while SimBio extends this functionality to CRNs, including support for the Systems Biology Markup Language (SBML). Poincaré and SimBio are developed as pure Python packages enabling users to easily extend their simulation capabilities by writing new or leveraging other Python packages. Moreover, this does not compromise the performance, as code can be just-in-time compiled with Numba. Our benchmark tests using curated models from the BioModels repository demonstrate that these tools may provide a potentially superior performance advantage compared to other existing tools. In addition, to ensure a user-friendly experience, our packages use standard typed modern Python syntax that provides a seamless integration with integrated development environments. Our Python-centric approach significantly enhances code analysis, error detection, and refactoring capabilities, positioning Poincaré and SimBio as valuable tools for the modeling community.

**Availability and implementation:**

Poincaré and SimBio are released under the MIT license. Their source code is available on GitHub (https://github.com/maurosilber/poincare and https://github.com/hgrecco/simbio) and can be installed from PyPI or conda-forge.

## 1 Introduction

Chemical reaction networks (CRNs) are a fundamental concept of modeling in numerous fields including systems biology, biochemistry, chemical engineering and epidemiology. They comprised a set of chemical species or biological entities and a set of reactions that mediate transformations between them. These systems can be simulated through multiple approaches: deterministic ordinary differential equations (ODEs) to model macroscopic behavior, stochastic differential equations (SDEs) to model microscopic fluctuations, and jump processes (Gillespie-like simulations) to account for the discreteness of populations. Instead of directly writing the equations for each of these formulations, which is error-prone and difficult to reuse, these models can be *defined* in a higher-level description that can be *translated* into equations for the different types of simulations and, then, *solved* numerically.

Several tools already exist to *define*, *translate*, and *solve* CRNs. BioSimulators.org (Shaikh *et al.* 2022), a registry of simulation tools, lists at least 15 softwares categorized under Python including COPASI (Hoops *et al.* 2006), Tellurium (Choi *et al.* 2018), and PySB (Lopez *et al.* 2013). COPASI is a standalone software with a graphical user interface (GUI) that is widely used for its user-friendly interface and comprehensive features. It also includes Python bindings, BASICO (Bergmann 2023), that allow advanced scripting. Tellurium is a Python-based modeling environment that uses a C++ library called libRoadRunner (Welsh *et al.* 2023) in the backend to translate and solve models. PySB is a Python library that created a domain-specific language (DSL) using standard Python to define models, which are then translated to ODEs using a Perl library called BioNetGen (Harris *et al.* 2016).

One limitation of these tools is their extensibility from Python. As they wrap libraries in other languages for defining, translating and/or solving models, these steps cannot be altered or easily inspected from Python. While they enable model definition and running simulations via Python scripts, they cannot fully leverage Python’s extensive package ecosystem. For example, COPASI and Tellurium do not allow the use of solvers defined in other Python packages, and adding new integrators requires working with C++. In particular, the step that translates into equations is not exposed by any of these tools. As such, it is not possible to apply custom optimizations to the equations or use automatic differentiation packages such as JAX (Bradbury *et al.* 2018) to compute the model’s jacobian.

Another challenge is the way models are defined. Many tools support the SBML (Hucka *et al.*) as an exchange format, a *de facto* standard for CRNs that defines species, parameters and reactions between species. As writing SBML directly is impractical, Tellurium uses a DSL called Antimony (Smith *et al.* 2009) for defining models. DSLs allows to reuse the same code in different programming environments, but are not recognized by default in integrated development environments (IDEs) and, therefore, they cannot provide syntax highlighting, code completion, refactoring, and static analysis. For Antimony, an extension providing these capabilities was developed for Visual Studio Code, but its maintenance could be a demanding task for the systems biology community. In the case of PySB, using Python’s dynamic nature, it developers designed a DSL within Python. To save keystrokes, it uses the global scope to create species and parameters, without explicitly assigning them to Python variables or to the model, but this approach is not fully compatible with IDEs, affecting the development experience.

To overcome these limitations, we developed poincaré and SimBio, open-source Python packages for defining, translating and solving systems. Poincaré allows one to define differential equation systems using variables, parameters and constants, and assigning rate equations to variables. For defining CRNs, SimBio builds on top of poincaré providing species and reactions that keep track of stoichiometries. Both are focused on providing an ergonomic experience to end-users by integrating well with IDEs and static analysis tools through the use of standard modern Python syntax. Moreover, since they are coded in pure Python, each step from model definition, translation to equations or solving can be extended or debugged from Python. Being the first-ever pure Python packages for systems modeling, they offer extensive extensibility, from simple tasks like reusing integrators defined in other packages, to complex ones like altering the compilation process to leverage some structure in the equations. For example, using a for-loop in the compiled equations could improve the runtime performance if there is some repetitive structure in the system, as happens in spatial modeling. The models built using these packages can be introspected to create other representations, such as graphs connecting species and/or reactions, or tables with parameters or equations. Furthermore, they have a modular architecture with a clear separation of concerns, making it easier to maintain or to contribute new code, which is beneficial for developers and maintainers. We showcased the reliability of these tools by benchmarking them against the simulation results from other tools. We also highlighted the substantial performance improvements our tools offer, as this is crucial for construction and simulation of models of whole cells and organisms, which necessitate the simulation of significantly large-scale models.

## 2 Results

Modular code architecture makes code reusable, extensible, and easier to maintain. Therefore, we split the code into three Python packages: symbolite, to create symbolic expressions; poincaré, to define dynamical systems; and simbio, to define CRNs and interface with systems biology standards such as SBML. These are pure Python packages with standard dependencies from the PyData scientific stack such as NumPy (Harris *et al.* 2020) and pandas (McKinney 2010). They are published in the Python package index (PyPI), where links to the source code and documentation hosted in GitHub can be found, and can be easily installed with pip install simbio, which installs symbolite and poincare as dependencies.

Symbolite is a lightweight symbolics package to create algebraic mathematical expressions. Unlike SymPy (Meurer *et al.* 2017), a widely used Python library for symbolic mathematics, it only provides the building of an expression tree which can be inspected and compiled to various backends. Symbolite is designed to facilitate the integration of new backends. Currently, we have implementations for NumPy (Harris *et al.* 2020); Numba (Lam *et al.* 2015), a just-in-time (JIT) compiler to LLVM; SymPy (Meurer *et al.* 2017); and JAX (Bradbury *et al.* 2018), a library that support automatic differentiation and compilation to graphical processing units (GPUs) and tensor processing units (TPUs).

### 2.1 Versatile modeling and simulation of dynamical systems with Poincaré

**Figure 1.**
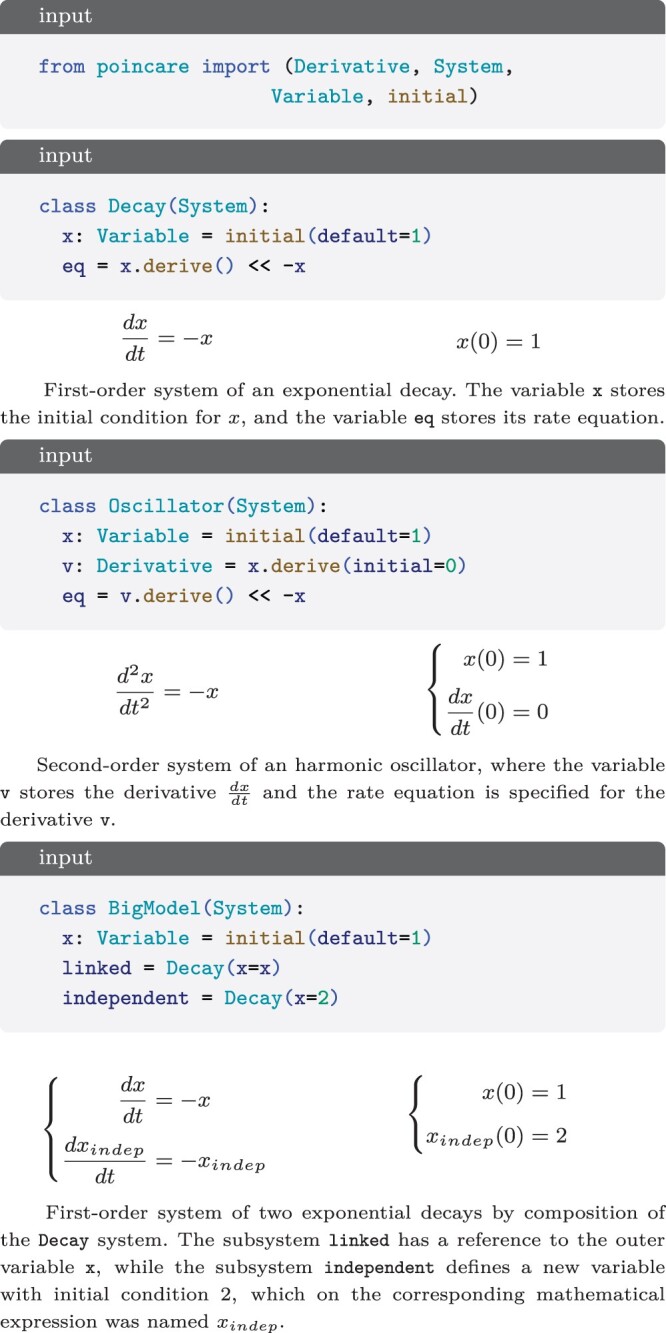
Code and corresponding mathematical expressions for different systems.

Poincare is a package to define and simulate dynamical systems. Based on Python immutable dataclasses, it provides a System class, where one can define Constants; which can be numbers or refer to other constants; Parameters, which can be numbers or time-dependent expressions; Variables, which represent the state of the system and must be provided with an initial condition; and create equations linking a variable’s derivative with an expression (Fig. 1a). It also allows to create an Independent variable to define nonautonomous systems, and define higher-order systems by assigning an initial condition to a Derivative (Fig. 1b).

Within the constraints imposed by Python’s current typing and static analyzers, we define models utilizing Python (data)classes such that we can benefit from IDEs’ autocomplete and refactoring capabilities. This design offers several advantages:

1. The variable name to which a component is assigned can be automatically saved in the component for introspection (i.e. Oscillator.x.name == “x”).
2. It provides a namespace that allows to easily define multiple independent models in the same script.
3. It allows IDEs to provide autocomplete and refactoring capabilities (Oscillator.<TAB> shows x, v, and eq).
4. It allows creation of instances that can be composed into a bigger model (Fig. 1b).

**Figure 2.**
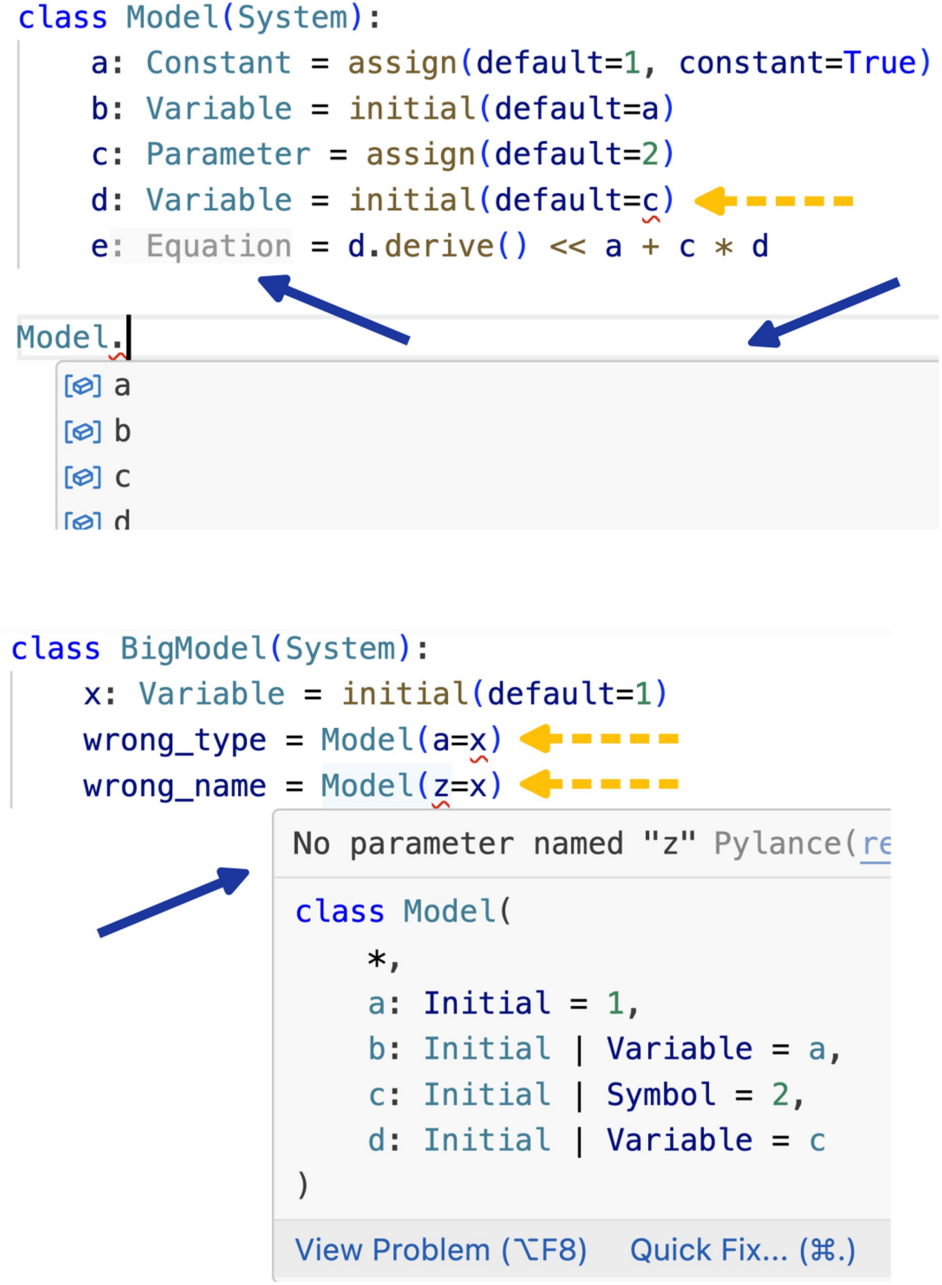
Screenshots of Visual Studio Code showing tooltips (solid blue arrows) and highlighted type errors (dashed yellow arrows). Above, we show that a, a Constant assigned with assign(…, constant=True), can be used for Variable b’s initial condition. Instead, it is flagged as a type error (red underlining) when using c, a Parameter, for Variable d’s initial condition, The IDE automatically recognizes e as an Equation, and provides autocompletion of the Model’s components. A tooltip is shown when composing models (solid blue arrow, below), which show the expected variables and their default values. The IDE highlights wrong names (z is not a name in Model) and mismatched types (x is Variable and a must be a number or a Constant).

For this last point, IDEs that support dataclass_transform (De Bonte and Traut 2021) can provide a tooltip with the expected signature (Fig. 2). This requires the use of type annotations, which play a more significant role in static type checking as they can help to identify errors before running the code. For instance, to parameterize the initial conditions of variables we have to use a Constant. If we try to use a Parameter, which could be a time-dependent expression, it is flagged as a type error (Fig. 2).

**Figure 3.**
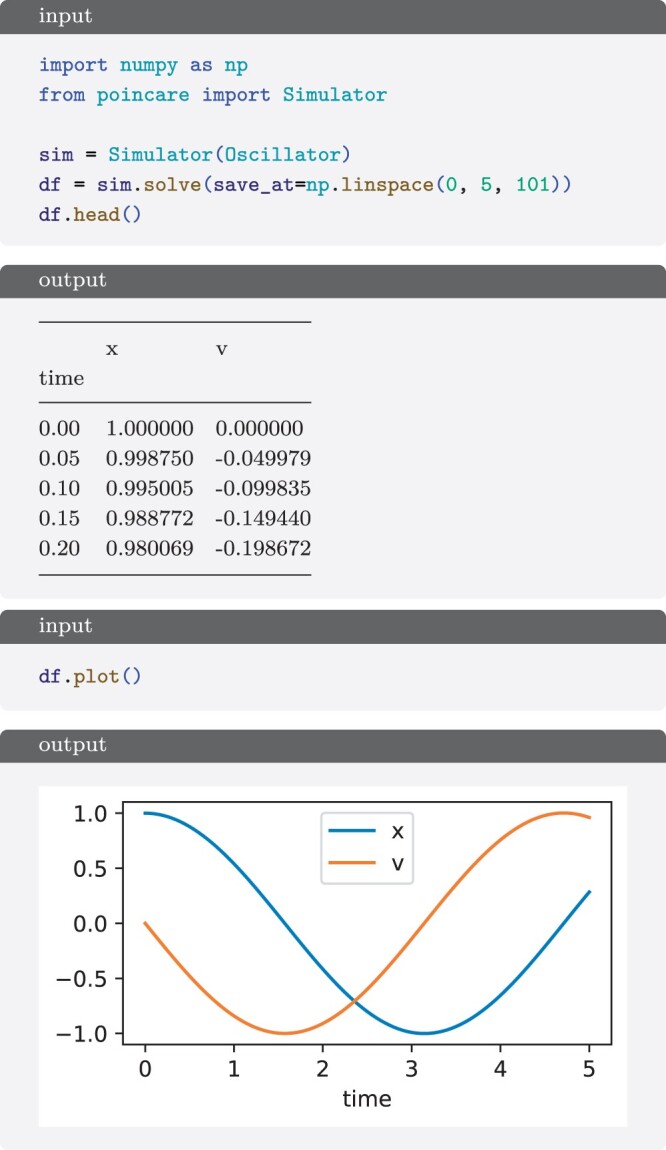
Simulation of the Oscillator system from Fig. 1b. The output is a pandas. DataFrame with a column for each variable and the time as index. It is inspected and plotted with the pandas methods.

To simulate a system, we created a Simulator instance (Fig. 3) that translates the model into the right-hand side (RHS) equations and interfaces with solvers wrapping the output in a pandas. DataFrame, which can be easily plotted with the standard plot method. Currently, it only supports translating into first-order ODEs, but it would be possible to add support for SDEs or delay differential equations (DDEs). By default, it uses numpy as a backend, and uses the LSODA solver from scipy. This can be easily switched to other solvers or backends, such as numba.

### 2.2 Extensible definition of reaction networks using SimBio

**Figure 4.**
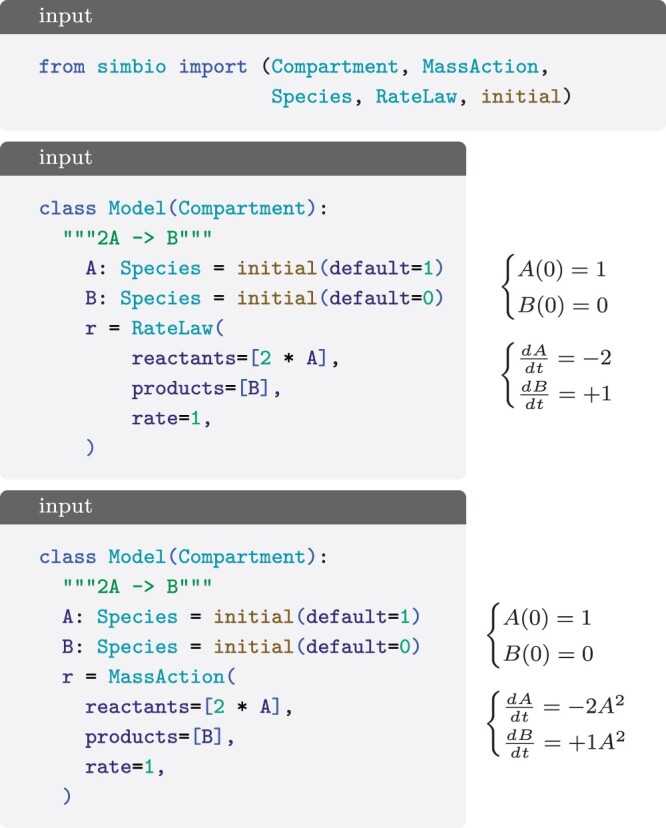
A reaction system for species *A* and *B* with initial conditions 1 and 0, respectively. A single reaction transforming 2*A* into *B* is saved in variable r. The rate 1 is specified directly for RateLaw, and is proportional to the reactants for MassAction.

For the CRNs, our focus is on first-order differential equations that describe the rate of change of species. SimBio simplifies the definition of these network models by introducing Species, and RateLaw, a construct that converts reactant species into product species taking into account the stoichiometry (Fig. 4). In addition, SimBio features MassAction, a subclass of RateLaw, that intuitively incorporates reactants and their stoichiometry into the rate law (Fig. 4).

**Figure 5.**
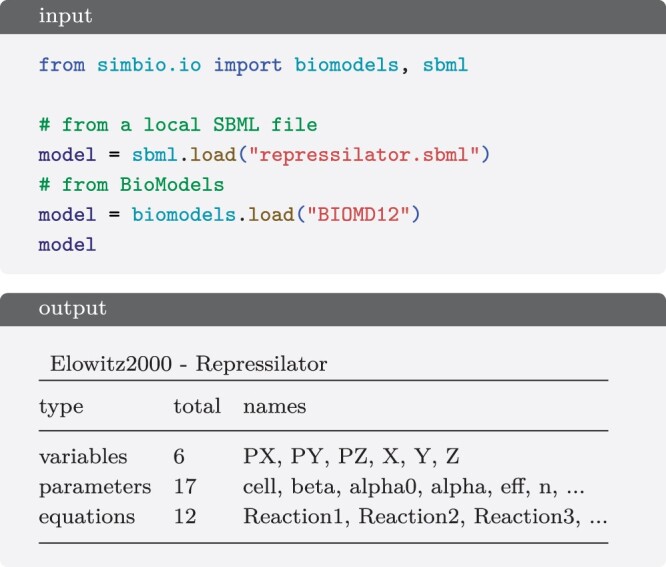
Creation of a model from a local SBML file or from one uploaded to BioModels.

Several commonly used reactions are predefined as MassAction subclasses, such as MichaelisMenten (S+E↔ES→P+E) and its approximate form without the intermediate species *ES*, and it is also simple to implement used-defined ones as subclasses of RateLaw or MassAction. In addition, SimBio supports importing models from SBML, and downloading them directly from BioModels (Malik-Sheriff *et al.* 2020) (Fig. 5). Work is in progress to support exporting to SBML and add more SBML features. Currently SBML unsupported features include algebraic rules, constraints, events, reactions with the fast attribute or with math stoichiometry, units and compartments with size different from 1.

### 2.3 Reproducibility and performance

To evaluate SimBio’s reproducibility, we used the SBML test suite (Hucka et al., 2017), which provides a set of SBML models and the expected result of a simulation. Excluding models that use SBML features not yet supported by SimBio, every simulation returned correct results within the solver tolerances.

To evaluate SimBio’s performance, we selected SBML models from the curated section of BioModels (Malik-Sheriff *et al.* 2020). Among the first 250 models, we considered the 117 that used supported SBML features. We ran simulations on a MacBook Air with M2 CPU using Python 3.11.8, COPASI v4.42.284 (with BasiCO v0.58) with the LSODA solver, RoadRunner v2.5.0 (with Tellurium v2.2.10) with the comparable CVODE solver, and SimBio v0.3.2 with the LSODA solver. For SimBio, we considered three variants: NumPy (v1.26.4) backend and scipy (v1.12.0)’s LSODA solver, Numba (v0.59.0) backend and scipy’s LSODA solver, Numba backend and numbalsoda’s (v0.3.5) LSODA solver. In all cases, we used a relative tolerance of 10^–6^ and absolute tolerances of 10^–9^. We measured two simulation stages: an initial cold run that includes the reading of the SBML model and subsequent warm runs.

**Figure 6.**
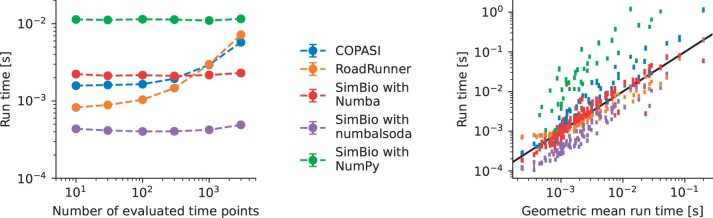
Performance of different softwares to solve models from the curated section of BioModels. (left) Run time for the model BIOMD3 as a function of the number of output points. (right) Run time for different models for 300 output points, using the geometric mean of the different softwares to order them. Each point corresponds to the median of 20 runs, with a negligible error-bar given by the interquartile range.

For COPASI and Tellurium, we noted that its runtime depended on the number of intermediate evaluation points returned to the user (Fig. 6, left). We speculate that this is due to memory allocation and data transfer in the Python bindings, as the number of total function evaluations (i.e. including those performed by the integrator stepper) is around 3000 and therefore much larger than most part of the *x*-axis. For SimBio, its NumPy backend can be orders of magnitude slower than both COPASI and RoadRunner (Fig. 6, right). Nevertheless, switching to the numba backend, which JIT compiles the RHS equations, puts it on par with them. Another speed-up in the runtime can be had by switching the LSODA scipy solver for a more efficient numbalsoda implementation that avoids calling into the Python interpreter between each of the integration steps. A user might have to consider the trade-off between compilation and run times, as the compilation of the RHS code might take longer than the runtime itself, and not be worth it for running the model only once. For the models considered, amortizing the compilation time required from 2 upto 200 runs.

## 3 Discussion

In this article, we introduced a suite of Python packages we developed for defining and simulating dynamical systems and CRNs. These packages are deeply integrated with IDEs, enabling code analysis tools to identify errors prior to execution and assist in refactoring and code completion. We adopted standard modern Python syntax to ensure seamless IDE integration, supported by the extensive Python community.

Our approach differs from previous tools in that both the model definition and its compilation into an ODE function are entirely Python-based. This approach simplifies the development of various simulation methods, including performance enhancements that exploit specific model structures. Importantly, being Python-based does not compromise performance compared to C/C++ tools, as the RHS functions can be JIT compiled using Numba.

The inclusion of SBML support facilitates the effortless reuse of models created by the systems biology community, along with the vast collection of public models hosted in the BioModels repository. The modular architecture of these packages facilitates their reuse, enhancement, and extension by the wider Python community. Therefore, it should be also easy to integrate with existing infrastructure such a BioSimulators.org (Shaikh *et al.* 2022), or combine with other packages like SimService (Sego 2024) to build more complex simulations. For instance, an individual from outside the systems biology field could contribute a stochastic integrator to poincaré, which would then be available in SimBio. This clear separation of concerns also makes the packages more comprehensible, lowering the barrier for contributing improvements or new features. Such an architecture ensures their maintainability and ongoing development well into the future.

## Data Availability

Source code repositories, example, and documentation for these packages can be found at https://github.com/maurosilber/poincare and https://github.com/hgrecco/simbio.
